# Mental and Physical Health Among Danish Transgender Persons Compared With Cisgender Persons

**DOI:** 10.1001/jamanetworkopen.2025.7115

**Published:** 2025-04-24

**Authors:** Dorte Glintborg, Jens-Jakob Kjer Møller, Katrine Hass Rubin, Øjvind Lidegaard, Guy T’Sjoen, Mie-Louise Julie Ørsted Larsen, Malene Hilden, Louise Lehmann Christensen, Marianne Skovsager Andersen

**Affiliations:** 1 Department of Endocrinology, Odense University Hospital, Odense, Denmark; 2 Institute of Clinical Research, University of Southern Denmark, Odense, Denmark; 3 OPEN–Open Patient Data Explorative Network, Odense University Hospital, Odense, Denmark; 4 Research Unit OPEN, Department of Clinical Research, University of Southern Denmark, Odense, Denmark; 5 Department of Gynaecology, Fertility and Obstetrics, Rigshospitalet, University of Copenhagen, Copenhagen, Denmark; 6 Department of Clinical Medicine, University of Copenhagen, Copenhagen, Denmark; 7 Department of Endocrinology and Center for Sexology and Gender, Ghent University Hospital, Ghent, Belgium; 8 Centre for Gender Identity, Department of Gynaecology, Rigshospitalet, University of Copenhagen, Copenhagen, Denmark

## Abstract

**Importance:**

Mental and somatic health is often impaired among transgender persons. Studies regarding coexisting mental and somatic health outcomes among transgender persons are limited.

**Objective:**

To assess health diagnoses and medicine use among transgender persons compared with cisgender controls.

**Design, Setting, and Participants:**

This register-based national cohort study included data from January 1, 2000, to December 31, 2021. Transgender persons were included on the first date of receipt of a transgender identity contact code. Controls included 10 age-matched cisgender men (n = 5) and women (n = 5) for each transgender person. Statistical analyses were conducted from September to December 2024.

**Main Outcomes and Measures:**

The main outcomes were *International Statistical Classification of Diseases and Related Health Problems, Tenth Revision* diagnosis codes and medicine use in a 5-year period up to the first date of transgender contact code for most commonly occurring mental and physical illnesses. The main outcomes were determined after data collection.

**Results:**

The cohort included 3812 transgender persons (1993 transmasculine persons [52.3%] with a median age of 19 years [IQR, 15-24 years] and 1819 transfeminine persons [47.7%] with a median age of 23 years [IQR, 19-33 years]) and 38 120 cisgender controls. The odds for a mental health diagnosis was up to 12 times higher among transgender persons compared with cisgender controls. Among transmasculine and transfeminine persons, neurotic, stress-related disorders (transmasculine: adjusted odds ratio [AOR], 4.70 [95% CI, 4.02-5.50]; transfeminine: AOR, 5.27 [95% CI, 4.28-6.49]); developmental disorders, including autism (transmasculine: AOR, 11.67 [95% CI, 8.85-15.39]; transfeminine: AOR, 9.39 [95% CI, 7.05-12.50]); mood (affective) disorders (transmasculine: AOR, 5.41 [95% CI, 4.32-6.77]; transfeminine: AOR, 5.61 [95% CI, 4.16-7.57]); and behavioral disorders (transmasculine: AOR, 4.50 [95% CI, 3.61-5.62]; transfeminine: AOR, 4.15 [95% CI, 3.19-5.39]) were the most frequent mental health diagnoses compared with cisgender controls of the opposite sex at birth. Transmasculine persons had higher odds for somatic diagnosis codes of diabetes (AOR, 2.00 [95% CI, 1.12-3.56]), asthma (including chronic obstructive lung disease; AOR, 1.40 [95% CI, 1.06-1.85]), injury and poisoning (AOR, 1.28 [95% CI, 1.15-1.41]), and pain (AOR, 1.29 [95% CI, 1.12-1.49]) compared with control cisgender women. Among transfeminine persons, somatic diagnosis codes of infection (AOR, 1.68 [95% CI, 1.33-2.13]), anemia (AOR, 3.08 [95% CI, 1.36-6.97]), diabetes (AOR, 1.95 [95% CI, 1.25-3.05]), sleep apnea (AOR, 3.41 [95% CI, 1.84-6.31]), and pain (AOR, 1.31 [95% CI, 1.08-1.58]) were more frequent compared with control cisgender men. Transgender persons had higher use of psychopharmacologic medicine, antacids, and laxatives compared with cisgender controls (transmasculine persons vs control cisgender women, antipsychotics: AOR, 6.20 [95% CI, 5.07-7.59]; hypnotics-sedatives: AOR, 4.45 [95% CI, 3.78-5.23]; antacids: AOR, 1.25 [95% CI, 1.07-1.45]; and laxatives: AOR, 1.53 [95% CI, 1.17-1.99]; transfeminine persons vs control cisgender men, antipsychotics: AOR, 4.74 [95% CI, 3.92-5.74]; hypnotics-sedatives: AOR, 3.01 [95% CI, 2.53-3.57]; and antacids: AOR, 1.32 [95% CI, 1.12-1.56]). Mental health diagnoses and use of psychopharmacologic drugs were coexisting with somatic diagnoses and use of drugs for somatic diseases.

**Conclusions and Relevance:**

This cohort study of Danish transgender persons and cisgender controls found significantly higher risks for mental and somatic health diagnoses among transgender persons. Coexistence of mental health outcomes and somatic health outcomes among transgender persons could be associated with stress encountered due to belonging to a gender identity or sexual orientation minority group; mental and physical morbidity should be considered an integrated part of transgender care.

## Key Points

**Question:**

What are the mental and somatic health outcomes among Danish transgender persons?

**Findings:**

This national register-based cohort study comprising 3812 Danish transgender persons and 38 120 cisgender controls found significantly higher odds for mental and somatic health diagnoses among transgender persons. Mental health diagnoses and use of psychopharmacologic drugs coexisted with somatic diagnoses and use of drugs for somatic diseases.

**Meaning:**

This study suggests that higher mental and physical morbidity could be associated with stress of belonging to a gender minority group and should be considered as an integrated part of transgender care.

## Teaser

This cohort study assesses physical and mental health diagnoses and medicine use among transgender persons compared with cisgender controls in Denmark.

## Introduction

The term *transgender* describes persons whose gender identity differs from their sex assigned at birth.^1^ The prevalence of transgender identity is estimated to be approximately 1%.^2,3^ Gender-affirming care in Denmark follows international guidelines^1,3^ and is conducted at highly specialized national public centers. Equal access to transgender care in Denmark and access to highly validated register data^4^ make it possible to map transgender health in a national large-scale setting.

Having a transgender identity is associated with legal discrimination in many countries, and it is well established that transgender persons have higher levels of mental and physical morbidities compared with cisgender controls.^5,6,7^ Minority stress (high levels of stress encountered by individuals belonging to a gender identity or sexual orientation minority group) is associated with adverse mental and physical health outcomes among transgender persons^8,9,10,11,12^ and is also associated with unhealthy lifestyle factors, hypercholesterolemia, higher blood pressure, and higher cardiovascular risk.^8,9^ Interventions to reduce stigma among transgender persons are therefore central for medical practice and health care policies.^13^

Diagnoses of depression and anxiety among transgender persons are more than 3-fold higher compared with cisgender controls.^6,14,15^ The prevalence of autism spectrum disorder is up to 26% higher among transgender persons than cisgender persons, but the mechanism for this higher risk is undetermined.^16^ Transgender persons have higher risks of cardiovascular disease (CVD),^9,12,17,18^ asthma, and lung diseases,^5,19^ whereas data on autoimmune diseases are nonuniform.^13,20^ Few previous studies have investigated the coexistence of multiple mental and physical health outcomes in large transgender cohorts.^7,21,22^ Most recently, the All of Us research program^7^ investigated 12 common health conditions among 30 763 persons of sexual or gender minorities (including 3687 gender diverse or transgender persons) with a median age range of older than 30 years and reported that anxiety, depression, and HIV diagnoses were more prevalent among people of sexual or gender minorities compared with cisgender heterosexual controls, whereas the occurrences of CVD, kidney disease, diabetes, and hypertension were lower. Transgender persons had higher mortality due to external causes^21^ and nonnatural causes of death.^22^ Possible associations between mental and physical health outcomes among transgender persons remain to be further elucidated. This study assesses mental and physical health among transgender persons seeking transgender care compared with cisgender controls.

## Methods

The study design was a register-based cohort study, and we followed the Strengthening the Reporting of Observational Studies in Epidemiology (STROBE) reporting guideline for cohort studies.^23^ In Denmark, a social security number is issued to citizens at birth or at immigration, which links data from Danish registers at an individual level.^4^ The National Patient Register includes information on psychiatric hospitals and outpatient contacts since 1995.^4^ The National Prescription Registry contains a complete record of the Anatomical Therapeutic Chemical (ATC) codes, dates of drug dispensed, and numbers of drug packets for all prescriptions filled at Danish pharmacies since 1995.^4^ The Danish Income Statistics Register includes the income of anyone economically active in Denmark.^24^ The Civil Registration System includes data on sex, age, date of death, and emigration since 1968.^4^ No approval was necessary from the local ethics committee or institutional review board on register studies according to Danish law.^4^ Data were anonymized, and no informed consent was needed from study participants. The study complied with the Declaration of Helsinki.^25^ The Data Protection Agency and Statistics Denmark approved the study with details published with OPEN ID.^26^

### Study Population

The study population included persons aged 3 years or older with an *International Statistical Classification of Diseases and Related Health Problems, Tenth Revision* (*ICD-10*) diagnosis code of transgender from January 1, 2000, to December 31, 2021, which would imply contact with a Danish center of gender identity. During 2000 to 2017, the diagnosis codes F64* (“transsexual, gender identity disorder”) were used,^27^ and from 2018 onward, the diagnosis code DZ768E* (“contact regarding gender identity condition”) was added. The index date (date of study inclusion) was the first date with a transgender diagnosis within the study period. Persons were required to have a valid Danish address on the index date.

### Controls

Five age-matched controls of the same sex at birth and 5 age-matched controls of the other sex at birth were included for each transgender person. Controls were assigned the index date of their matched transgender person. The controls were alive and had a valid Danish address at the index date of their respective matched transgender person. Each control could be included only once.

*Assigned sex at birth* in the transgender study cohort was determined as the earliest recorded Danish Civil Registration System–encoded sex. Transgender persons were divided into transfeminine persons (persons assigned male sex at birth) and transmasculine persons (persons assigned female sex at birth). Misclassification of assigned sex at birth could occur among immigrating persons who had undergone sex change abroad and among persons with transgender diagnosis or legal sex change before 2000. Therefore, persons with surgical codes of salpingo-oophorectomy and/or hysterectomy were defined as transmasculine persons and persons with diagnosis codes of penis amputation and/or orchiectomy were defined as transfeminine persons, which resulted in correction of assigned sex for 5 individuals (eFigure in Supplement 1).

### Definition of Study Parameters

Study outcomes included diagnoses related to hospital contacts and medicine prescribed and dispensed to members of the study cohort in a 5-year period prior to the index date. From all diagnoses related to hospital contacts and prescribed medicine for the transgender cohort and controls, we identified the 10 most prevalent mental diagnosis categories, the 10 most prevalent somatic diagnosis categories (*ICD-10* codes), and the 17 most frequently prescribed medicines according to prescribed ATC drug groups (ATC codes). Based on the most prevalent diagnoses, we defined the following study outcomes:

- *ICD-10* diagnoses (eTable 1 in Supplement 1) for mental and behavioral disorders: 1, organic mental disorder; 2, psychoactive substance use; 3, schizophrenia and delusional disorders; 4, mood (affective); 5, neurotic, stress related; 6, eating disorders; 7, adult personality; 8, mental retardation; 9, developmental disorder, autism; and 10, behavioral disorder.
- *ICD-10* diagnoses (eTable 1 in Supplement 1) for somatic disease: 1, infections; 2, neoplasms; 3, anemia; 4, diabetes; 5, sleep apnea; 6, airway infections; 7, asthma (including chronic obstructive pulmonary disease [COPD]); 8, noninfectious enteritis, colitis; 9, injury and poisoning; and 10, pain.
- ATC codes for medicine use: 1, analgesics; 2, antiepileptics; 3, antipsychotics; 4, anxiolytics; 5, hypnotics and sedatives; 6, antidepressants; 7, anti–attention-deficit/hyperactivity disorder (ADHD) medications; 8, antacids; 9, laxatives; 10, antidiabetics; 11, antithrombotic agents; 12, diuretics, antihypertensives, and lipid-modifying agents; 13, sex hormones; 14, thyroid therapy; 15, antibiotics; 16, airway, asthma, and COPD medications; and 17, antihistamines and dermal corticosteroids.

Results for mental health diagnosis codes and use of psychopharmacologic drugs in the study cohort have been published previously.^6^

### Covariates

Age was calculated at the index date. Ethnicity was extracted from the Civil Registration System and divided into Danish and non-Danish (including immigrants and descendants). Ethnicity was included as a confounder in the adjusted models. Employment was measured through the Employment Classification Module^24^ and divided into 2 categories: employed (wage earner, self-employed, retired, or student [including elementary school]) and unemployed (out of work, early retirement, and unknown).

Personal income was extracted from the Income Statistics Register^24^ in the calendar year of the index date (or the first calendar year prior to the index date in case of missing income information) and included information on the total annual income (included salary, retirement benefits, welfare payment, remuneration, and company profits). Income was categorized into tertiles (low, middle, and high) within age groups, with 5- to 7-year intervals based on 54 274 individuals (the 41 932 individuals from the study populations, along with 1112 individuals who had legally changed their sex, matched with 11 220 control individuals). Income was included as a confounder in the adjusted models.

### Statistical Analysis

Statistical analyses were conducted from September to December 2024. Baseline characteristics of transgender persons were presented as frequencies for categorical variables and compared using the χ^2^ test. Study outcomes included those that occurred 5 years before and until the index date. Conditional logistic regression models were applied to compare transgender persons with cisgender control women and men, respectively, using each transgender person’s identity number as a matched group variable. Unadjusted odds ratios (ORs) and adjusted ORs (AORs) of the dichotomous presence of diagnoses and redeemed medicine prescriptions for transgender persons 5 years before study inclusion compared with cisgender controls were calculated for income tertiles and ethnicity.

Data management and data analyses were conducted using StataMP, version 18.0 (StataCorp LLC). Network graphs were created in RStudio using the igraph library with R, version 4.3.2 (R Project for Statistical Computing). All *P* values were from 2-sided tests and results were deemed statistically significant at *P* < .05.

## Results

The transgender cohort included 3812 individuals; 1993 (52.3%) were transmasculine persons, and 1819 of 3812 (47.7%) were transfeminine persons (eFigure in Supplement 1). The median age at the index date was 19 years (IQR, 15-24 years) for transmasculine persons and 23 years (IQR, 19-33 years) for transfeminine persons (Table 1). The control group comprised 38 120 cisgender individuals. The prevalence of persons of non-Danish origin was significantly lower among transgender persons than cisgender controls of the same and other birth sex. Transgender persons had a higher prevalence of low-tertile income and were more often unemployed compared with cisgender controls of same and other birth sex.^6^

**Table 1.** Characteristics of Danish Transgender Cohort (N = 3812) and Controls (N = 38 120) 5 Years Before the Index Date^a^

Characteristic	Transmasculine persons (n = 1993)^b^	Cisgender controls for transmasculine persons		Transfemine persons (n = 1819)^b^	Cisgender controls for transfeminine persons	
		Women (n = 9965)	Men (n = 9965)		Men (n = 9095)	Women (n = 9095)
Age, median (IQR), y	19 (15-24)	19 (15-24)	19 (15-24)	23 (19-33)	23 (19-33)	23 (19-33)
Ethnicity, No. (%)						
Danish	1820 (91.3)	8551 (85.8)	8564 (85.9)	1617 (88.9)	7812 (85.9)	7801 (85.8)
Non-Danish	173 (8.7)	1414 (14.2)	1401 (14.1)	202 (11.1)	1283 (14.1)	1294 (14.2)
Employed, No. (%)						
No	436 (27.3)	782 (9.8)	695 (8.7)	579 (38.8)	970 (12.9)	1055 (14.1)
Yes	1164 (72.8)	7220 (90.2)	7318 (91.3)	914 (61.2)	6531 (87.1)	6447 (85.9)
Income, No. (%)						
Low tertile	931 (46.7)	3456 (34.7)	3500 (35.1)	1003 (55.1)	3044 (33.5)	3152 (34.7)
Middle tertile	725 (36.4)	3607 (36.2)	3169 (31.8)	513 (28.2)	2697 (29.7)	3272 (36.0)
High tertile	337 (16.9)	2902 (29.1)	3296 (33.1)	303 (16.7)	3354 (36.9)	2671 (29.4)

^a^ Baseline characteristics included ethnicity, personal income, and use of gender-affirming hormone therapy in transgender cohort and controls. The transgender cohort was divided between transgender men and transgender women. Statistical analyses were performed for transgender men vs control women, transgender men vs control men, transgender women vs control men, and transgender women vs control women.

^b^ Transgender persons and controls were matched for age. All comparisons between transgender persons and controls of same and other birth sex have *P* < .05 except age as the groups were age matched.

### *ICD-10* Diagnoses

**Figure 1.**
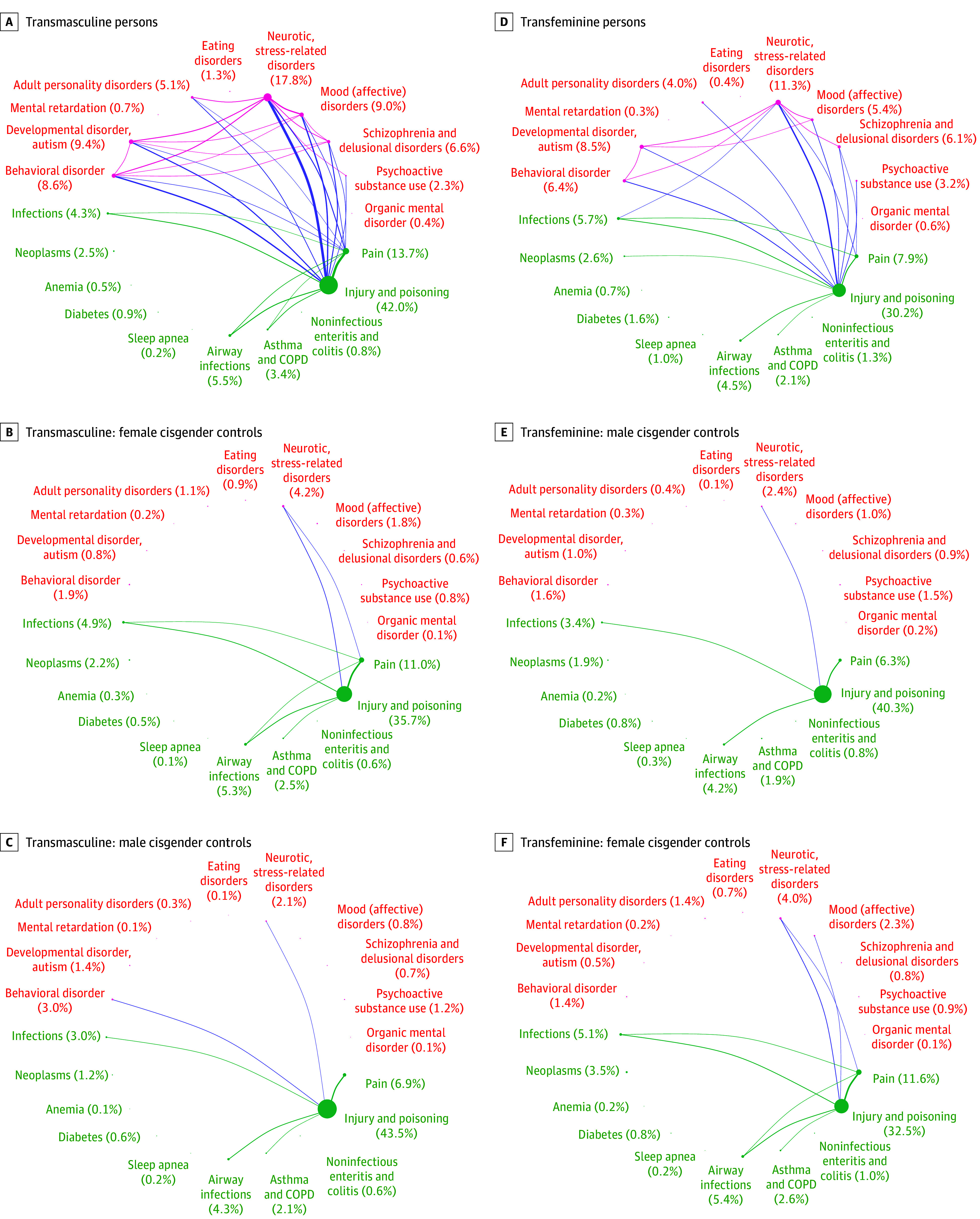
*International Statistical Classification of Diseases and Related Health Problems, Tenth Revision* Diagnosis Codes Among Transgender Persons and Cisgender Controls Baseline: date of first transgender diagnosis. Connections are drawn when more than 1% of the cohort has the combination of 2 comorbidities. COPD indicates chronic obstructive pulmonary disease. Larger circles indicate higher disease or drug prevalence, and thicker lines indicate a more frequent co-occurrence between diseases and drugs.

Among transmasculine persons compared with cisgender control women, all diagnoses of mental and behavioral disorders (except eating disorders) were more frequent in the transgender cohort. Neurotic, stress-related disorders (354 of 1993 [17.8%] vs 420 of 9965 [4.2%]; AOR, 4.70 [95% CI, 4.02-5.50]); developmental disorders, including autism (188 of 1993 [9.4%] vs 82 of 9965 [0.8%]; AOR, 11.67 [95% CI, 8.85-15.39]); mood (affective) disorders (179 of 1993 [9.0%] vs 178 of 9965 [1.8%]; AOR, 5.41 [95% CI, 4.32-6.77]); and behavioral disorders (171 of 1993 [8.6%] vs 189 of 9965 [1.9%]; AOR, 4.50 [95% CI, 3.61-5.62]) were the most frequent mental health diagnoses among transmasculine persons (Table 2; eTable 2 in Supplement 1). Diagnosis codes of diabetes (17 of 1993 [0.9%] vs 48 of 9965 [0.5%]; 2.00 [95% CI, 1.12-3.56]), asthma (including COPD; 68 of 1993 [3.4%] vs 246 of 9965 [2.5%]; AOR, 1.40 [95% CI, 1.06-1.85]), injury and poisoning (838 of 1993 [42.0%] vs 3560 of 9965 [35.7%]; AOR, 1.28 [95% CI, 1.15-1.41]), and pain (273 of 1993 [13.7%] vs 1099 of 9965 [11.0%]; AOR, 1.29 [95% CI, 1.12-1.49]) were more frequent among transmasculine persons compared with cisgender control women (Table 2; Figure 1; eTable 2 in Supplement 1).

**Table 2.** Diagnosis Codes Among Transgender Persons and Cisgender Controls

Diagnoses	*ICD-10* code	Transmasculine persons vs cisgender controls^a^				Transfeminine persons vs cisgender controls^a^			
		Cisgender women		Cisgender men		Cisgender men		Cisgender women	
		OR (95% CI)	AOR (95% CI)	OR (95% CI)	AOR (95% CI)	OR (95% CI)	AOR (95% CI)	OR (95% CI)	AOR (95% CI)
Mental and behavioral disorders									
Organic mental disorders	F00-F09	11.26 (2.93-43.27)^b^	7.72 (1.65-36.21)^c^	5.65 (1.99-16.08)^b^	4.08 (1.33-12.57)^c^	3.54 (1.62-7.73)^c^	4.25 (1.77-10.23)^b^	4.82 (2.09-11.10)^b^	4.99 (2.11-11.83)^b^
Psychoactive substance use	F10-F19	2.89 (2.00-4.17)^b^	2.84 (1.94-4.14)^b^	1.92 (1.36-2.71)^b^	1.76 (1.23-2.51)^b^	2.17 (1.59-2.96)^b^	2.14 (1.55-2.96)^b^	3.55 (2.53-4.97)^b^	3.10 (2.19-4.39)^b^
Schizophrenia and delusional disorders	F20-F29	11.12 (8.16-15.15)^b^	10.23 (7.46-14.02)^b^	9.64 (7.19-12.94)^b^	8.35 (6.14-11.36)^b^	6.79 (5.09-9.06)^b^	7.00 (5.18-9.45)^b^	8.39 (6.19-11.39)^b^	7.13 (5.19-9.79)^b^
Mood (affective) disorders	F30-F39	5.55 (4.46-6.91)^b^	5.41 (4.32-6.77)^b^	12.70 (9.66-16.68)^b^	10.71 (8.08-14.21)^b^	5.69 (4.25-7.62)^b^	5.61 (4.16-7.57)^b^	2.42 (1.89-3.10)^b^	2.08 (1.61-2.69)^b^
Neurotic, stress-related disorders	F40-F48	4.92 (4.22-5.73)^b^	4.70 (4.02-5.50)^b^	10.44 (8.70-12.52)^b^	9.37 (7.78-11.29)^b^	5.38 (4.39-6.59)^b^	5.27 (4.28-6.49)^b^	3.07 (2.57-3.68)^b^	2.71 (2.25-3.27)^b^
Eating disorders	F50	1.42 (0.90-2.22)	1.40 (0.89-2.22)	15.98 (7.19-35.53)^b^	15.43 (6.88-34.59)^b^	6.67 (2.31-19.21)^b^	5.48 (1.44-20.87)^c^	0.60 (0.29-1.26)	0.35 (0.15-0.82)
Adult personality disorders	F60-F69	4.68 (3.55-6.16)^b^	4.36 (3.29-5.78)^b^	18.02 (11.92-27.23)^b^	15.00 (9.82-22.92)^b^	10.74 (7.15-16.13)^b^	10.80 (7.01-16.62)^b^	3.03 (2.25-4.07)^b^	2.50 (1.82-3.44)^b^
Mental retardation	F70-F79	4.07 (1.92-8.59)^b^	3.95 (1.70-9.14)^b^	5.58 (2.52-12.33)^b^	5.41 (2.35-12.46)^b^	1.30 (0.53-3.20)	1.18 (0.47-2.95)	1.36 (0.55-3.36)	1.28 (0.51-3.19)
Developmental disorder, including autism	F84	12.72 (9.69-16.69)^b^	11.67 (8.85-15.39)^b^	7.38 (5.87-9.27)^b^	6.64 (5.26-8.38)^b^	9.56 (7.25-12.60)^b^	9.39 (7.05-12.50)^b^	18.66 (13.33-26.12)^b^	17.20 (12.15-24.35)^b^
Behavioral disorders	F90-F98	4.96 (3.99-6.16)^b^	4.50 (3.61-5.62)^b^	3.09 (2.54-3.77)^b^	2.89 (2.36-3.53)^b^	4.28 (3.32-5.52)^b^	4.15 (3.19-5.39)^b^	5.04 (3.88-6.55)^b^	4.67 (3.56-6.11)^b^
Infections	A00-B99, N30	0.87 (0.69-1.10)	0.89 (0.70-1.13)	1.46 (1.14-1.87)^c^	1.49 (1.16-1.92)^c^	1.70 (1.35-2.14)^b^	1.68 (1.33-2.13)^b^	1.12 (0.90-1.39)	1.11 (0.88-1.39)
Neoplasms	C00-D48	1.10 (0.80-1.51)	1.13 (0.82-1.56)	2.19 (1.56-3.09)^b^	2.28 (1.61-3.23)^b^	1.36 (0.98-1.90)	1.37 (0.98-1.92)	0.72 (0.53-0.99)	0.74 (0.54-1.02)
Anemias	D55-D64	2.02 (0.96-4.24)	2.06 (0.97-4.39)	10.27 (3.49-30.26)^b^	10.14 (3.41-30.11)^b^	3.31 (1.57-6.96)^c^	3.08 (1.36-6.97)^b^	4.12 (1.91-8.91)^b^	3.90 (1.72-8.82)^b^
Diabetes	E10-E14	1.77 (1.02-3.08)^c^	2.00 (1.12-3.56)^c^	1.55 (0.90-2.67)	1.60 (0.92-2.79)	1.97 (1.27-3.06)^c^	1.95 (1.25-3.05)^c^	2.14 (1.38-3.32)^b^	2.20 (1.40-3.46)^b^
Sleep apnea	G47.3	2.50 (0.75-8.30)	3.23 (0.84-12.42)	1.18 (0.40-3.50)	1.16 (0.38-3.53)	3.20 (1.78-5.74)^b^	3.41 (1.84-6.31)^b^	5.45 (2.84-10.47)^b^	5.59 (2.87-10.89)^b^
Airway infections	J00-J06, J18, J20-J22, J30-J39, J96	1.03 (0.83-1.28)	1.04 (0.84-1.28)	1.28 (1.03-1.58)^b^	1.35 (1.09-1.69)^b^	1.08 (0.84-1.38)	1.10 (0.86-1.41)	0.82 (0.65-1.05)	0.85 (0.66-1.08)
Asthma and COPD	J40-J47, J96, R06	1.40 (1.06-1.84)^c^	1.40 (1.06-1.85)^c^	1.68 (1.27-2.22)	1.72 (1.30-2.28)^c^	1.14 (0.80-1.62)	1.08 (0.75-1.55)	0.81 (0.57-1.14)	0.74 (0.52-1.06)
Noninfective enteritis and colitis	K50-K52	1.23 (0.70-2.18)	1.20 (0.68-2.15)	1.19 (0.68-2.10)	1.26 (0.71-2.23)	1.52 (0.95-2.44)	1.58 (0.98-2.56)	1.27 (0.80-2.01)	1.21 (0.75-1.95)
Injury and poisoning	S00-S99, T00-T32, T36-T50, K70	1.31 (1.19-1.45)^b^	1.28 (1.15-1.41)^b^	0.94 (0.85-1.04)^c^	0.96 (0.87-1.06)	0.64 (0.57-0.71)^b^	0.65 (0.58-0.72)^b^	0.90 (0.81-1.00)	0.94 (0.84-1.05)
Pain	R07, R10, R51, R52, M25.5, M79	1.28 (1.11-1.48)^b^	1.29 (1.12-1.49)^b^	2.15 (1.85-2.50)^b^	2.16 (1.85-2.51)^c^	1.28 (1.06-1.55)^c^	1.31 (1.08-1.58)^c^	0.65 (0.54-0.78)^b^	0.66 (0.55-0.80)^b^

Abbreviations: AOR, adjusted odds ratio; *ICD-10*, *International Statistical Classification of Diseases and Related Health Problems, Tenth Revision*; OR, odds ratio.

^a^ Transgender persons and cisgender controls of same and other birth sex using ORs. Adjusted ORs are adjusted for income tertiles and ethnicity.

^b^ *P* < .001.

^c^ *P* < .05.

Among transfeminine persons vs control cisgender men, all diagnoses of mental and behavioral disorders were more frequent among the transgender cohort, except mental retardation. Neurotic, stress-related disorders (206 of 1819 [11.3%] vs 214 of 9095 [2.4%]; AOR, 5.27 [95% CI, 4.28-6.49]); developmental disorders, autism (154 of 1819 [8.5%] vs 94 of 9095 [1.0%]; AOR, 9.39 [95% CI, 7.05-12.50]); and behavioral disorders (117 of 1819 [6.4%] vs 149 of 9095 [1.6%]; AOR, 4.15 [95% CI, 3.19-5.39]) were the most frequent mental health diagnoses among transfeminine persons (Table 2; eTable 2 in Supplement 1). Diagnosis codes of infection (103 of 1819 [5.7%] vs 313 of 9095 [3.4%]; AOR, 1.68 [95% CI, 1.33-2.13]), anemia (12 of 1819 [0.7%] vs 19 of 9095 [0.2%]; AOR, 3.08 [95% CI, 1.36-6.97]), diabetes (29 of 1819 [1.6%] vs 76 of 9095 [0.8%]; AOR, 1.95 [95% CI, 1.25-3.05]), sleep apnea (19 of 1819 [1.0%] vs 31 of 9095 [0.3%]; AOR, 3.41 [95% CI, 1.84-6.31]), and pain (144 of 1819 [7.9%] vs 573 of 9095 [6.3%]; AOR, 1.31 [95% CI, 1.08-1.58]) were more frequent among transfeminine persons than control cisgender men, whereas injury and poisoning (550 of 1819 [30.2%] vs 3661 of 9095 [40.3%] AOR, 0.65 [95% CI, 0.58-0.72]) were less frequent.

### Medicine Use

Transmasculine persons had higher use of psychopharmacologic medicine, antacids, and laxatives compared with cisgender control women (antipsychotics: AOR, 6.20 [95% CI, 5.07-7.59]; hypnotics-sedatives: AOR, 4.45 [95% CI, 3.78-5.23]; antacids: AOR, 1.25 [95% CI, 1.07-1.45]; and laxatives: AOR, 1.53 [95% CI, 1.17-1.99]) (Table 3). Transfeminine persons also had higher use of these drug classes compared with cisgender control men (antipsychotics: AOR, 4.74 [95% CI, 3.92-5.74]; hypnotics-sedatives: AOR, 3.01 [95% CI, 2.53-3.57]; and antacids: AOR, 1.32 [95% CI, 1.12-1.56]).

**Table 3.** Medicine Prescriptions in Transgender Persons and Controls

Medicine	ATC code	Transmasculine persons vs cisgender controls^a^				Transfeminine persons vs cisgender controls^a^			
		Cisgender women		Cisgender men		Cisgender men		Cisgender women	
		OR (95% CI)	AOR (95% CI)	OR (95% CI)	AOR (95% CI)	OR (95% CI)	AOR (95% CI)	OR (95% CI)	AOR (95% CI)
Analgesics	N02, M01	1.07 (0.96-1.20)	1.06 (0.95-1.19)	1.88 (1.68-2.10)^b^	1.92 (1.71-2.16)^b^	0.86 (0.76-0.97)^c^	0.86 (0.76-0.98)^c^	0.52 (0.46-0.58)^b^	0.53 (0.47-0.59)^b^
Antiepileptics	N03A	2.35 (1.81-3.04)^b^	2.30 (1.76-2.99)^b^	2.94 (2.25-3.84)^b^	2.58 (1.96-3.40)^b^	1.95 (1.51-2.51)^b^	1.87 (1.44-2.42)^b^	1.45 (1.13-1.86)^c^	1.27 (0.98-1.63)
Antipsychotics	N05A	6.33 (5.20-7.70)^b^	6.20 (5.07-7.59)^b^	6.38 (5.27-7.72)^b^	5.46(4.48-6.65)^b^	4.72 (3.92-5.68)^b^	4.74 (3.92-5.74)^b^	4.41 (3.68-5.29)^b^	3.91 (3.24-4.72)^b^
Anxiolytics	N05B	2.94 (2.30-3.75)^b^	2.94 (2.29-3.76)^b^	4.82 (3.70-6.27)^b^	4.36 (3.33-5.71)^b^	2.39 (1.88-3.03)^b^	2.20 (1.72-2.82)^b^	1.26 (1.01-1.58)^c^	1.16 (0.92-1.46)
Hypnotics and sedatives	N05C	4.54 (3.87-5.32)^b^	4.45 (3.78-5.23)^b^	5.67 (4.83-6.67)^b^	5.20 (4.40-6.14)^b^	3.07 (2.59-3.64)^b^	3.01 (2.53-3.57)^b^	2.12 (1.80-2.49)^b^	1.95 (1.65-2.30)^b^
Antidepressants	N06A	4.88 (4.24-5.63)^b^	4.85 (4.19-5.62)^b^	8.73 (7.48-10.20)^b^	7.55 (6.42-8.87)^b^	4.76 (4.09-5.54)^b^	4.68 (4.01-5.47)^b^	2.45 (2.14-2.81)^b^	2.12 (1.84-2.44)^b^
Anti-ADHD medication	N06B	4.80 (3.90-5.90)^b^	4.43 (3.59-5.48)^b^	2.58 (2.15-3.10)^b^	2.30 (1.90-2.78)^b^	3.18 (2.58-3.92)^b^	3.09 (2.49-3.84)^b^	4.65 (3.71-5.82)^b^	3.98 (3.14-5.03)^b^
Antacids	A02	1.25 (1.07-1.45)^c^	1.25 (1.07-1.45)^c^	2.18 (1.86-2.56)^b^	2.13 (1.81-2.50)^b^	1.34 (1.14-1.58)^b^	1.32 (1.12-1.56)^b^	0.77 (0.66-0.91)	0.75 (0.64-0.88)
Laxatives	A06	1.54 (1.18-2.00)^b^	1.53 (1.17-1.99)^c^	2.31 (1.75-3.04)^b^	2.23 (1.69-2.94)^b^	1.39 (1.01-1.91)^c^	1.38 (1.00-1.90)	1.07 (0.79-1.46)	1.08 (0.79-1.47)
Antidiabetics	A10	1.81 (1.22-2.68)^c^	1.90 (1.27-2.84)^c^	2.15 (1.43-3.21)^b^	2.10 (1.39-3.17)^b^	1.33 (0.93-1.91)	1.31 (0.91-1.88)	1.29 (0.91-1.84)	1.26 (0.88-1.80)
Antithrombotic agents	B01	1.01 (0.62-1.64)	1.00 (0.62-1.63)	1.43 (0.86-2.38)	1.34 (0.80-2.25)	1.10 (0.77-1.55)	1.09 (0.77-1.55)	1.38 (0.98-1.95)	1.38 (0.98-1.95)
Diuretics, antihypertensives, lipid-modifying medications	C03, C07-C10	1.27 (1.00-1.62)	1.26 (0.99-1.62)	2.18 (1.68-2.82)^b^	1.99 (1.52-2.59)^b^	1.66 (1.34-2.06)^b^	1.69 (1.36-2.10)^b^	1.12 (0.92-1.37)	1.07 (0.87-1.31)
Sex hormones	G03	0.32 (0.28-0.36)^b^	0.26 (0.23-0.30)^b^	391.8 (214.9-714.4)^b^	502.9 (267.4-945.9)^b^	25.82 (16.18-41.20)^b^	23.10 (14.16-37.69)^b^	0.04 (0.03-0.05)^b^	0.04 (0.03-0.05)^b^
Thyroid therapy	H03	1.31 (0.88-1.95)	1.21 (0.80-1.83)	5.97 (3.57-10.00)^b^	5.40 (3.16-9.24)^b^	1.68 (0.89-3.17)	1.48 (0.75-2.90)	0.34 (0.19-0.60)^b^	0.29 (0.16-0.52)^b^
Antibiotics	J, P, D01, D06, D10, P02, S01A	0.61 (0.55-0.68)^b^	0.57 (0.51-0.64)^b^	1.17 (1.05-1.30)^c^	1.19 (1.07-1.33)^c^	0.84 (0.76-0.94)	0.83 (0.74-0.93)^b^	0.42 (0.37-0.47)^b^	0.42 (0.37-0.47)^b^
Airway, asthma, and COPD medication	R01, R03	1.17 (1.04-1.31)^c^	1.14 (1.01-1.28)^c^	1.28 (1.14-1.44)^b^	1.26 (1.12-1.41)^b^	1.06 (0.94-1.20)	1.08 (0.95-1.23)	0.84 (0.74-0.95)^c^	0.86 (0.75-0.97)^c^
Antihistamines and dermal corticosteroids	R06, D07	0.88 (0.79-0.98)	0.86 (0.78-0.96)	1.16 (1.04-1.28)^c^	1.16 (1.04-1.29)^c^	1.20 (1.08-1.34)^b^	1.22 (1.09-1.36)^b^	0.80 (0.72-0.89)^b^	0.81 (0.72-0.90)^b^

Abbreviations: ADHD, attention-deficit/hyperactivity disorder; AOR, adjusted odds ratio; ATC, Anatomical Therapeutic Chemical classification system; COPD, chronic obstructive airway disease; OR, odds ratio.

^a^ Transgender persons and cisgender controls of same and other birth sex using ORs. Adjusted ORs are adjusted for income tertiles and ethnicity.

^b^ *P* < .001.

^c^ *P* < .05.

Among transmasculine persons compared with control cisgender women, use of antiepileptics (90 of 1993 [4.5%] vs 201 of 9965 [2.0%]), antipsychotics (251 of 1993 [12.6%] vs 244 of 9965 [2.4%]), anxiolytics (113 of 1993 [5.7%] vs 214 of 9965 [2.1%]), hypnotics and sedatives (334 of 1993 [16.8%] vs 455 of 9965 [4.6%]), antidepressants (460 of 1993 [23.1%] vs 646 of 9965 [6.5%]), anti-ADHD medicine (186 of 1993 [9.3%] vs 215 of 9965 [2.2%]), antacids (253 of 1993 [12.7%] vs 1050 of 9965 [10.5%]), laxatives (78 of 1993 [3.9%] vs 260 of 9965 [2.6%]), antidiabetics (35 of 1993 [1.8%] vs 99 of 9965 [1.0%]), and airway, asthma, and COPD medication (475 of 1993 [23.8%] vs 2107 of 9965 [21.2%]) was more frequent among transmasculine persons (eTable 2 in Supplement 1). The use of sex hormones (555 of 1993 [27.8%] vs 4842 of 9965 [48.6%]), antibiotics (1395 of 1993 [70.0%] vs 7859 of 9965 [78.9%]), and antihistamines and dermal corticosteroids (625 of 1993 [31.4%] vs 3404 of 9965 [34.2%]) was less frequent among transmasculine persons than control cisgender women.

**Figure 2.**
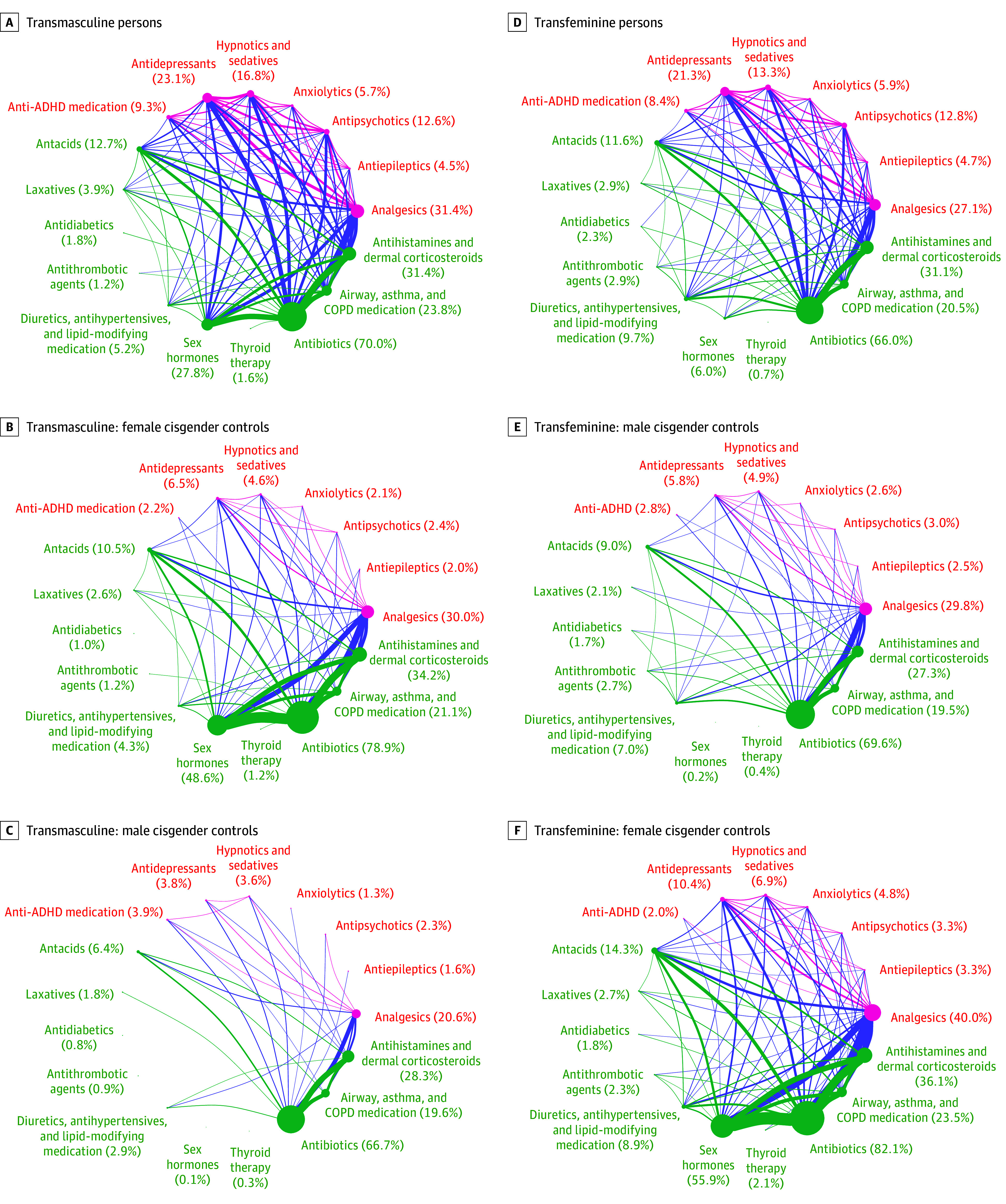
Medicine Prescriptions Among Transgender Persons and Cisgender Controls Baseline: date of first transgender diagnosis. Connections are drawn when more than 1% of the cohort has the combination of 2 comorbidities. ADHD indicates attention-deficit/hyperactivity disorder; and COPD, chronic obstructive pulmonary disease. Larger circles indicate higher disease or drug prevalence, and thicker lines indicate a more frequent co-occurrence between diseases and drugs.

Among transfeminine persons compared with control cisgender men, use of antiepileptics (85 of 1819 [4.7%] vs 225 of 9095 [2.5%]); antipsychotics (232 of 1819 [12.8%] vs 274 of 9095 [3.0%]); anxiolytics (107 of 1819 [5.9%] vs 238 of 9095 [2.6%]); hypnotics and sedatives (242 of 1819 [13.3%] vs 443 of 9095 [4.9%]); antidepressants (388 of 1819 [21.3%] vs 532 of 9095 [5.8%]); anti-ADHD medicine (152 of 1819 [8.4%] vs 258 of 9095 [2.8%]); antacids (211 of 1819 [11.6%] vs 818 of 9095 [9.0%]); laxatives (52 of 1819 [2.9%] vs 191 of 9095 [2.1%]); diuretic, antihypertensive, and lipid-lowering medicine (176 of 1819 [9.7%] vs 639 of 9095 [7.0%]); sex hormones (109 of 1819 [6.0%] vs 22 of 9095 [0.2%]); and antihistamines and dermal corticosteroids (566 of 1819 [31.1%] vs 2486 of 9095 [27.3%]) was more frequent among transfeminine persons (Table 3; Figure 2; eTable 2 in Supplement 1). Use of analgesics (493 of 1819 [27.1%] vs 2709 of 9095 [29.8%]) and antibiotics (1201 of 1819 [66.0%] vs 6333 of 9095 [69.6%]) was less frequent among transfeminine persons than control cisgender men. *ICD-10* diagnoses and medicine use among transgender persons compared with controls of the opposite birth sex are presented in Table 2, Table 3, and eTable 2 in Supplement 1.

The prevalence and coexistence of morbidity among transgender persons and control cisgender persons are shown in Figure 1 and Figure 2. Among transmasculine and transfeminine persons, diagnosis of injury and poisoning coexisted with most mental health diagnoses, and the highest degree of coexistence was seen with neurotic, stress-related disorders.

Medicine use was more frequent among transgender persons compared with cisgender controls, which was especially evident for transmasculine persons compared with male cisgender controls. Antibiotics were the most commonly prescribed medication among transmasculine and transfeminine persons, and use of antibiotics had a high degree of coexistence with medical treatment for mental health diagnoses; use of antihistamines, dermal corticosteroids, and airway medication; and asthma and COPD medication.

## Discussion

In this register-based cohort study, we investigated morbidity among 3812 transgender persons 5 years before contact with a Danish national center of gender identity. The study outcomes included a wide selection of mental and somatic diagnosis codes and medicine prescriptions. We found that transgender persons had higher mental and physical morbidity compared with cisgender controls. Furthermore, mental health diagnoses and use of psychopharmacologic drugs among transgender persons coexisted with somatic study outcomes and use of drugs for somatic diseases.

Among transmasculine and transfeminine transgender persons, we found that the AOR for mental and behavioral disorders (such as schizophrenia), delusional disorders, and developmental disorders (such as autism) were more than 10-fold higher compared with controls. Furthermore, transmasculine persons had higher odds for a diagnosis of diabetes, asthma or COPD, injury or poisoning, or pain compared with cisgender control women, whereas among transfeminine persons, diagnosis codes of infection, anemia, diabetes, sleep apnea, and pain were more frequent compared with control cisgender men. Among transgender persons, mental health diagnoses coexisted with diagnoses of injury and poisoning, pain, and infections. Use of psychopharmacologic agents coexisted with use of a wide range of drugs for treatment of somatic diseases, including antibiotics, analgesics, antihistamines and dermal corticosteroids, antacids, and airway, asthma, and COPD medications. These findings support a high degree of coexistence between several mental and somatic health outcomes among transgender persons.

Our finding of higher mental and somatic morbidity among transgender persons compared with cisgender controls expand the results from recent publications showing impaired mental health,^6^ higher risk of CVD,^18^ and a lower employment rate^28^ among Danish transgender persons compared with controls. However, we and other study groups have previously focused on selected and hypothesis-driven study outcomes,^6,17,18,20,29^ whereas fewer studies evaluated several study outcomes simultaneously.^7,13,21,22^ Poorer mental health among transgender persons is supported by studies showing that the overall OR for mental health outcomes was 5.00 among Danish transgender persons,^6^ and the prevalence of depression was approximately 50% among transgender persons.^6,30,31,32^ Furthermore, transgender persons had higher risks of injuries and death from external causes^21,22^ and higher risks of cardiovascular, pulmonary, and lifestyle-related diseases.^5,17,22^

The risk and mechanism for adverse somatic health outcomes among transgender cohorts can involve genetic, epigenetic, and lifestyle factors.^5,11,17,18,22^ Our finding of a high degree of coexistence between mental and physical health outcomes among transgender persons supports a common mechanism, such as minority stress, for these outcomes.^8^ The present study did not include questionnaire data regarding minority stress. Socioeconomic status and the employment rate, which are markers of minority stress,^33^ are lower among transgender persons compared with cisgender controls,^5,28,34,35,36^ and lower socioeconomic status is associated with a higher risk of experiencing health care discrimination.^37^ Furthermore, a lower employment rate is associated with mental health diagnoses,^28^ suicidal behavior,^15^ and depression^38^ among transgender persons and is associated with adverse physical health outcomes, such as obesity, CVD, and cancer.^28,39,40^ In our adjusted analyses, the AORs for health outcomes among transgender persons compared with cisgender controls were attenuated but remained significant. These findings support that even if minority stress is associated with mental and somatic health, other factors should be considered. In particular, the reason for a high prevalence of autism among transgender persons is unclarified.^41^ Autism spectrum disorder is a separate risk factor for comorbidities, such as type 2 diabetes, dyslipidemia, and CVD.^42^ Our findings are in close alignment with a recent large survey from New Zealand including 876 transgender participants showing a higher risk of hypertension, myocardial infarction, stroke, hypercholesteremia, and poor or fair general health among the transgender participants compared with controls^13^; higher transgender-related stigma was associated with risk of hypertension, hypercholesterolemia, and poor or fair health. A systematic review found substantial evidence supporting the association between minority stress and physical health outcomes, such as respiratory infection, immune response, HIV and AIDS, CVD, body mass index (calculated as weight in kilograms divided by height in meters squared), cortisol level, and cancer incidence.^8^ These results suggest a direction for future studies on pathways for health outcomes among transgender persons, and studies are needed on the physical health disparities faced by transgender persons.^13^ Our findings support the need for multifaceted support and clinical care for transgender persons, including addressing minority stress and aiming for stigma reduction as part of clinical and political interventions.

### Strengths and Limitations

This study has some strengths. An important strength is the access to nationwide data and the ability to include all persons with contact with national centers for gender identity in Denmark. A systematic psychological and somatic evaluation after referral to a center of gender identity could lead to diagnosis and treatment of study outcomes, and we defined baseline as 5 years before referral. The study outcomes spanned the full range of mental and somatic health and outcomes and were based on an unselected draft of diagnosis codes and medicine use in the study cohort.

The study also has some limitations. Each of the study outcomes consisted of a number of separate diagnoses, which could differ in cause and/or disease mechanism. The present study included results on baseline health before referral to a center of gender identity, and the study outcomes could be affected by receipt of gender-affirming care, including hormone treatment. The complexity of study outcomes did not allow for detailed prospective analyses, and further studies should investigate individual diagnoses in more detail and/or apply other methods to define study outcomes. Low levels of legal discrimination in Denmark could also affect study results.

Transgender identity was defined according to *ICD-10* codes; therefore, transgender persons and nonbinary persons not attending a public center for gender identity would not be included in the dataset. The wish for gender-affirming care is often the primary concern when meeting with a health care professional and seeking referral to a transgender clinic. Some transgender persons could have more severe psychiatric diseases or untreated mental and physical disease, which could delay referral for transgender care. In Denmark, a body mass index of more than 27 could limit access to several procedures of gender-affirming surgery at public hospitals, and some persons will consult private clinics for surgery. Therefore, the present study cohort represents a selected group of transgender persons. Self-identification of gender identity was not available in the present study, and the study cohort is expected to include gender nonbinary people. Sexual orientation is not binary,^43^ and sexual orientation was not part of the present study, nor were other minority groups.

## Conclusions

In this cohort study of 3812 Danish transgender persons and 38 120 cisgender controls, mental and physical morbidity was generally higher among transgender persons compared with cisgender controls. The high degree of coexistence between mental and physical health outcomes highlights the importance of a multifaceted approach to transgender persons, including addressing minority stress.

## Notes

Supplement 1. **eTable 1.** Description of *ICD-10* Study Outcomes **eTable 2.** Characteristics of Danish Transgender Cohort (N= 3,812) and Controls (N= 38,120) Including Diagnoses Codes and Medicine Prescriptions Five Years Before the Index Date **eFigure.** Flowchart of Study Cohort

Supplement 2.
Data Sharing Statement
