# Error in Figure 2

**DOI:** 10.1001/jamanetworkopen.2025.49290

**Published:** 2025-11-24

In the Original Investigation titled “Mental and Physical Health Among Danish Transgender Persons Compared With Cisgender Persons,”^1^ published April 24, 2025, panel C in Figure 2 was incorrectly labeled “Transfeminine: male cisgender controls” instead of “Transmasculine: male cisgender controls.” This article has been corrected.

## References

[1] Glintborg D, Møller JJK, Rubin KH, et al. Mental and physical health among Danish transgender persons compared with cisgender persons. JAMA Netw Open. 2025;8(4):e257115. doi:10.1001/jamanetworkopen.2025.7115 40272800 PMC12022810

