# Supplementary material for: Mental and Physical Health Among Danish Transgender Persons Compared With Cisgender Persons
Source: JAMA Netw Open. 2025 Apr 24;8(4):e257115. doi: 10.1001/jamanetworkopen.2025.7115 (PMC12022810; doi:10.1001/jamanetworkopen.2025.7115)
Supplement: Supplement 1. — eTable 1. Description of ICD-10 Study Outcomes eTable 2. Characteristics of Danish Transgender Cohort (N= 3,812) and Controls (N= 38,120) Including Diagnoses Codes and Medicine Prescriptions Five Years Before the Index Date eFigure. Flowchart of Study Cohort [file jamanetwopen-e257115-s001.pdf]

Supplementary Online Content

Glintborg D, Møller JJK, Rubin KH, et al. Mental and physical health among Danish transgender persons compared with cisgender persons. *JAMA Netw Open.* 2025;8(4):e257115. doi:10.1001/jamanetworkopen.2025.7115

eTable 1. Description of *ICD-10* Study Outcomes
eTable 2. Characteristics of Danish Transgender Cohort (N= 3,812) and Controls (N= 38,120) Including Diagnoses Codes and Medicine Prescriptions Five Years Before the Index Date
eFigure. Flowchart of Study Cohort

This supplementary material has been provided by the authors to give readers additional information about their work.

eTable 1. Description of *ICD-10* Study Outcomes

Diagnoses, *ICD-10*	Description
Mental and behavioral disorders	
Organic mental disorder (F00-F09)	Organic, including symptomatic, mental disorders.<br>Dementia in Alzheimer disease, vascular dementia, unspecified dementia<br>Personality and behavioural disorders due to brain disease, damage and dysfunction
Psychoactive substance use (F10-F19)	Mental and behavorial disorders due to use of alcohol, opioids, cannabinoids, Hypnotics and sedativess, cocaine, tobacco, drug use etc.
Schizophrenia and delusional (F20-F29)	Scizophrenia, persistent delusional disorders, acute psychotic disorders, skizoaffective disorders, nonorganic psychotic disorders.
Mood (affective)(F30-F39)	Manic episode, bipolar affective disorder, depressive episode, recurrent depressive disorder, persistent mood disorder.
Neurotic, stress related (F40-F48)	Neurotic, stress-related and somatoform disorders. Anxiety disorders, obsessive compulsive disorder, stress and adjustment disorder, dissociative disorder, somatoform disorder, neurotic disorder.
Eating disorders (F50)	Anorexia, bulimia.
Adult personality (F60-F69)	Personality disorder, habit and impulse disorder, gender identity disorder, disorders of sexual preference.
Mental retardation (F70-F79)	Mild, moderate, severe, profound mental retardation
Developmental disorder, autism (F84)	Pervasive developmental disorders, childhood autism, atypical autism, Asperger syndrome
Behavioral disorder (F90-F98)	Hyperkinetic disorder, conduct disorder, emotional disorder with onset specific to childhood, Tic disorder, other behavioural and emotional disorders with onset usually occurring in childhood and adolescence
Somatic diagnoses	
Infections (A00-B99, N30)	Certain infectious and parasitic diseases excluding influenza and other acute respiratory infections (J00-J22), N30 Cystitis
Neoplasms (C00-D48)	C00-C97: Malignant neoplasms, D00-D09 In situ neoplasms, D10-D36 Benign neoplasms D37-D48 Neoplasms of uncertain or unknown behaviour
Anemia (D55-D59, D60-D64)	D55-D59 Haemolytic anaemias, D60-D64 Aplastic and other anaemias
Diabetes mellitus (E10-E14)	Type 1 diabetes mellitus, type 2 diabetes mellitus, unspecified diabetes mellitus
Sleep apnea (G473)	Sleep apnoea

© 2025 Glintborg D et al. *JAMA Network Open.*

Airway infections (J00-J06, J18, J20-J22, J30-J39, J96)	J00-J06 Acute upper respiratory infections, J18 Pneumonia, organism unspecified, J20-22 Other acute lower respiratory infections, J30-J39 Other diseases of upper respiratory tract, J96 Respiratory failure, not elsewhere classified
Asthma, COPD (J40-J47, J96, R06)	J40-J47 Chronic lower respiratory diseases, J96 Respiratory failure, not elsewhere classified, R06, Abnormalities of breathing, stridor, hyperventilation
Noninfections enteritis, colitis (K50-K52)	K50-K52 Noninfective enteritis and colitis
Injury, poisoning (S00-S99, T00-T32, T36-T50, K70)	S00-S99 Injuries of head, neck, thorax… ankle foot, T00-T32 multiple injuries, trauma, burns T36-T50 Poisoning by drugs, medication, medicaments and biological substances, K70 Alcoholic liver disease
Pain (R07, R10, R51, R52, M255, M79)	R07 Throat and neck, R10 Abdominal and pelvic, R51 headache, R52 pain, not elsewhere specified, M255 joint, M79 Other soft tissue disorders, not elsewhere classified, myalgia, fibromyalgia, limb

ICD-10 codes of included study outcomes.

© 2025 Glintborg D et al. *JAMA Network Open.*

eTable 2. Characteristics of Danish Transgender Cohort (N= 3,812) and Controls (N= 38,120) Including Diagnoses Codes and Medicine Prescriptions Five Years Before the Index Date

	**Transmasculine persons** N=1,993	**Control Cisgender women** N=9,965	**Control Cisgender men** N=9,965	**Transfeminine persons** N=1,819	**Control Cisgender men** N=9,095	**Control Cisgender women** N=9,095
**ICD-10 diagnoses**						
Mental and behavioral disorders Organic mental disorder (F00-F09)	8 (0.4%)	6 (0.1%)	9 (0.1%)	11 (0.6%)	16 (0.2%)	12 (0.1%)
Psychoactive substance use (F10-F19)	46 (2.3%)	82 (0.8%)	122 (1.2%)	59 (3.2%)	140 (1.5%)	86 (0.9%)
Schizophrenia and delusional (F20-F29)	131 (6.6%)	62 (0.6%)	73 (0.7%)	111 (6.1%)	85 (0.9%)	71 (0.8%)
Mood (affective) (F30-F39)	179 (9.0%)	178 (1.8%)	78 (0.8%)	98 (5.4%)	90 (1.0%)	211 (2.3%)
Neurotic, stress related (F40-F48)	354 (17.8%)	420 (4.2%)	206 (2.1%)	206 (11.3%)	214 (2.4%)	366 (4.0%)
Eating disorders (F50)	25 (1.3%)	89 (0.9%)[x]	8 (0.1%)	8 (0.4%)	6 (0.1%)	66 (0.7%)
Adult personality (F60-F69)	101 (5.1%)	114 (1.1%)	30 (0.3%)	73 (4.0%)	34 (0.4%)	125 (1.4%)
Mental retardation (F70-F79)	13 (0.7%)	17 (0.2%)	12 (0.1%)	6 (0.3%)	23 (0.3%)[x]	22 (0.2%)[x]
Developmental disorder, autism (F84)	188 (9.4%)	82 (0.8%)	144 (1.4%)	154 (8.5%)	94 (1.0%)	48 (0.5%)
Behavioral disorder (F90-F98)	171 (8.6%)	189 (1.9%)	299 (3.0%)	117 (6.4%)	149 (1.6%)	127 (1.4%)
Infections (A00-B99, N30)	85 (4.3%)	485 (4.9%)[x]	295 (3.0%)	103 (5.7%)	313 (3.4%)	464 (5.1%)[x]
Neoplasms (C00-D48)	49 (2.5%)	224 (2.2%)[x]	115 (1.2%)	47 (2.6%)	176 (1.9%)[x]	319 (3.5%)
Anemia (D55-D59, D60-D64)	10 (0.5%)	25 (0.3%)[x]	5 (0.1%)	12 (0.7%)	19 (0.2%)	15 (0.2%)
Diabetes mellitus (E10-E14)	17 (0.9%)	48 (0.5%)	55 (0.6%)[x]	29 (1.6%)	76 (0.8%)	69 (0.8%)
Sleep apnea (G473)	4 (0.2%)	8 (0.1%)[x]	17 (0.2%)[x]	19 (1.0%)	31 (0.3%)	18 (0.2%)
Airway infections (J00-J06, J18, J20-J22, J30-J39, J96)	109 (5.5%)	529 (5.3%)[x]	433 (4.3%)	82 (4.5%)	382 (4.2%)[x]	493 (5.4%)[x]
Asthma, COPD (J40-J47, J96, R06)	68 (3.4%)	246 (2.5%)	205 (2.1%)	39 (2.1%)	172 (1.9%)[x]	240 (2.6%)[x]
Noninfections enteritis, colitis (K50-K52)	15 (0.8%)	61 (0.6%)[x]	63 (0.6%)[x]	23 (1.3%)	76 (0.8%)[x]	91 (1.0%)[x]
Injury, poisoning (S00-S99, T00-T32, T36-T50, K70)	838 (42.0%)	3,560 (35.7%)	4,334 (43.5%)[x]	550 (30.2%)	3,661 (40.3%)	2,954 (32.5%)[x]
Pain (R07, R10, R51, R52, M255, M79)	273 (13.7%)	1,099 (11.0%)	689 (6.9%)	144 (7.9%)	573 (6.3%)	1,058 (11.6%)
**ATC#**						
Analgesics (N02, M01)	625 (31.4%)	2,994 (30.0%)[x]	2,055 (20.6%)	493 (27.1%)	2,709 (29.8%)	3,641 (40.0%)

© 2025 Glintborg D et al. *JAMA Network Open.*

Antiepileptics (N03A)	90 (4.5%)	201 (2.0%)	161 (1.6%)	85 (4.7%)	225 (2.5%)	299 (3.3%)
Antipsykotics (N05A)	251 (12.6%)	244 (2.4%)	233 (2.3%)	232 (12.8%)	274 (3.0%)	296 (3.3%)
Anxiolytics (N05B)	113 (5.7%)	214 (2.1%)	132 (1.3%)	107 (5.9%)	238 (2.6%)	434 (4.8%)
Hypnotics and sedatives (N05C)	334 (16.8%)	455 (4.6%)	363 (3.6%)	242 (13.3%)	443 (4.9%)	630 (6.9%)
Antidepressants (N06A)	460 (23.1%)	646 (6.5%)	380 (3.8%)	388 (21.3%)	532 (5.8%)	950 (10.4%)
Anti ADHD (N06B)	186 (9.3%)	215 (2.2%)	386 (3.9%)	152 (8.4%)	258 (2.8%)	178 (2.0%)
Antacids (A02)	253 (12.7%)	1,050 (10.5%)	642 (6.4%)	211 (11.6%)	818 (9.0%)	1,305 (14.3%)
Laxatives (A06)	78 (3.9%)	260 (2.6%)	176 (1.8%)	52 (2.9%)	191 (2.1%)	243 (2.7%)[×]
Antidiabetics (A10)	35 (1.8%)	99 (1.0%)	84 (0.8%)	41 (2.3%)	157 (1.7%)[×]	161 (1.8%)[×]
Antithrombotic agents (B01)	24 (1.2%)	119 (1.2%)[×]	91 (0.9%)[×]	53 (2.9%)	248 (2.7%)[×]	205 (2.3%)[×]
Diuretics, anti hypertensives, lipid modifying (C03, C07, C08, C09, C10)	104 (5.2%)	431 (4.3%)[×]	287 (2.9%)	176 (9.7%)	639 (7.0%)	814 (8.9%)[×]
Sex hormones (G03)	555 (27.8%)	4,842 (48.6%)	11 (0.1%)	109 (6.0%)	22 (0.2%)	5,085 (55.9%)
Thyroid therapy (H03)	32 (1.6%)	124 (1.2%)[×]	28 (0.3%)	13 (0.7%)	39 (0.4%)[×]	187 (2.1%)
Antibiotics (J, P, D01, D06, D10, P02, S01A)	1,395 (70.0%)	7,859 (78.9%)	6,642 (66.7%)	1,201 (66.0%)	6,333 (69.6%)	7,468 (82.1%)
Airway, asthma, COPD (R01, R03)	475 (23.8%)	2,107 (21.1%)	1,955 (19.6%)	372 (20.5%)	1,774 (19.5%)[×]	2,134 (23.5%)
Antihistamines, dermal corticosteroids (R06, D07)	625 (31.4%)	3,404 (34.2%)	2,822 (28.3%)	566 (31.1%)	2,486 (27.3%)	3,285 (36.1%)

Baseline characteristics including ICD-10 diagnosis codes and medication history five years before the index date.
Transgender cohort divided in transgender men and transgender women.

Statistical analyses performed in transgender men vs. control women, transgender men vs. control men, transgender women vs. control men, and in transgender women vs. control women
All P-values <0.05 except [×]: P>0.05.

ATC: Anatomical Therapeutic Chemical Classification System, ICD-10: International codes of diagnoses, IQR: Interquartile range
#≥2 redemptions of medicine

© 2025 Glintborg D et al. *JAMA Network Open.*

eFigure. Flowchart of Study Cohort

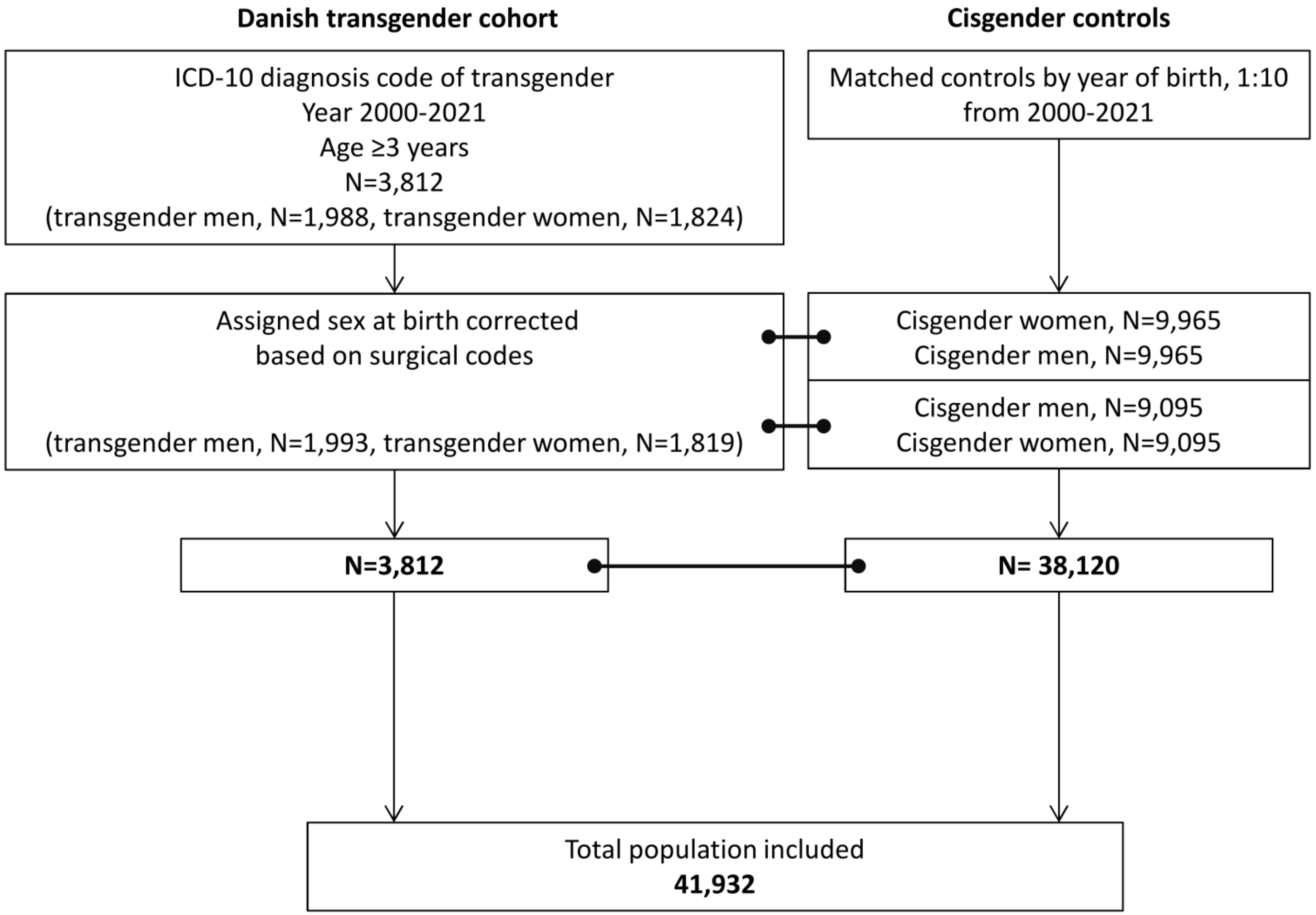

© 2025 Glintborg D et al. *JAMA Network Open.*

Flowchart of included study participants divided into transgender men and transgender women.

Controls included 5 age-matched cisgender persons of same and other birth sex.

© 2025 Glintborg D et al. *JAMA Network Open.*
