# Supplementary material for: Mental and Physical Health Among Danish Transgender Persons Compared With Cisgender Persons
Source: JAMA Netw Open. 2025 Apr 24;8(4):e257115. doi: 10.1001/jamanetworkopen.2025.7115 (PMC12022810; doi:10.1001/jamanetworkopen.2025.7115)
Supplement: Supplement 2. — Data Sharing Statement [file jamanetwopen-e257115-s002.pdf]

Data Sharing Statement

Glintborg. Mental and Physical Health Among Danish Transgender Persons Compared With Cisgender Persons. *JAMA Netw Open*. Published April 24, 2025.
doi:10.1001/jamanetworkopen.2025.7115

Data
Data available: No

Additional Information
Explanation for why data not available: Danish legislation does not allow sharing of the dataset as individual level data are protected according to GDPR (general data protection regulation) rules. Authors can be contacted regarding summarized results, if needed. The coding library is available upon request.
